# Comparison of outcomes between modified double-flanged sutureless scleral fixation and conventional sutured scleral fixation

**DOI:** 10.1038/s41598-024-66762-y

**Published:** 2024-07-12

**Authors:** Jinsoo Kim, Phil Young Lee, Min Seon Park, Bum-Joo Cho, Soonil Kwon

**Affiliations:** 1 Department of Ophthalmology, Hallym University Sacred Heart Hospital, Hallym University College of Medicine, 22 Gwanpyeong-Ro 170Beon-Gil, Dongan-Gu, Anyang, 14068 Gyeonggi-Do Korea; 2 Department of Ophthalmology, Veterans Health Service Medical Center, Seoul, Korea

**Keywords:** Lens diseases, Retinal diseases

## Abstract

This retrospective study aimed to compare the outcomes of modified double-flanged sutureless scleral fixation versus sutured scleral fixation. Medical records of 65 eyes from 65 patients who underwent double-flanged scleral fixation (flange group) or conventional scleral fixation (suture group) between 2021 and 2022 were reviewed. Visual and refractive outcomes, as well as postoperative complications, were compared 1, 2, and 6 months after surgery. We included 31 eyes in the flange group and 34 eyes in the suture group. At 6 months postoperatively, the flange group showed better uncorrected visual acuity (0.251 ± 0.328 vs. 0.418 ± 0.339 logMAR, *P* = 0.041) and a smaller myopic shift (− 0.74 ± 0.93 vs. − 1.33 ± 1.15 diopter, *P* = 0.007) compared to the suture group. The flange group did not experience any instances of iris capture, while the suture group had iris capture in 10 eyes (29.4%; *P* < 0.001). In the flange group, all intraocular lenses remained centered, whereas in the suture group, they were decentered in 8 eyes (23.5%; *P* = 0.005). The double-flanged technique not only prevented iris capture and decentration of the intraocular lens but also reduced myopic shift by enhancing the stability of the intraocular lens.

## Introduction

In cases of the absence of capsular or zonular support, such as aphakia, dislocated posterior chamber intraocular lens (IOL), or crystalline lens subluxation resulting from trauma or complications of cataract surgery, the implantation of an IOL into the capsular bag is precluded^1,2^. Various techniques have been used to place an IOL in an eye without capsular support, including angle-supported anterior chamber IOLs, iris-claw IOLs, sutured scleral-fixated IOLs, fibrin glue-assisted intrascleral-fixated IOLs, and flanged intrascleral-fixated IOLs^3–8^.

Being the oldest method employed, angle-supported anterior chamber IOLs necessitate a peripheral iridotomy and a wide scleral tunnel, which carries a risk of corneal endothelial dysfunction^2,4^. Similarly, the retropupillary iris-claw IOLs, such as the ARTISAN Aphakia IOL (Ophthec BV, Groningen, Netherlands), require a wide corneal incision or scleral tunnel of at least 5.4 mm, but offer the advantage of preserving the iridocorneal angle^5^. However, both techniques have limitations in their application to compromised irides^1,9^. On the other hand, scleral-fixated IOLs can be used in eyes with damaged irides and can help avoid complications such as corneal endothelial damage^2,9^. Since Lewis popularized the ab externo method in 1991^3^, conventional sutured scleral fixation has been primarily used^1,7^, but this surgical method has the disadvantage of prolonged duration due to its technical complexity^5,6,8^. To simplify the procedure and mitigate the risks for suture-related complications in sutured scleral-fixated IOLs, Yamane et al^6^. introduced the flange method of sutureless intrascleral haptic fixation. This technique involves cauterizing the end of the haptics to create end-bulb flanges, which has contributed to its widespread adoption due to its simplicity and expedited execution^10^. Nevertheless, there still remains a risk of iris capture, IOL tilt, as well as a learning curve to overcome the difficulty of haptic and needle docking, which can lead to haptic kinking or breakage^10,11^.

Recently, inspired by the Yamane technique, Canabrava et al. developed a double-flanged (four-flanged) knotless intrascleral fixation technique cauterizing a 5–0 or 6–0 polypropylene thread with a four-haptic foldable IOL to reduce the risk of tilt in two-point scleral-fixated IOLs and suture-related complications^7,12^. Until now, there is no consensus regarding which surgical technique is the most effective or safest for IOL implantation in the absence of capsular support^9^. To the best of our knowledge, no previous study has compared the outcomes of the double-flanged technique with another surgical technique. Therefore, this study aimed to compare the modified double-flanged technique and conventional sutured scleral fixation.

## Results

### Patient characteristics

A total of 65 eyes from 65 patients were included in the study, consisting of 19 eyes (29.2%) with aphakia, 37 eyes (56.9%) with a dislocated IOL, and 9 eyes (13.8%) with a subluxated crystalline lens (Table 1). The mean postoperative follow-up period of all patients was 14.0 ± 6.6 months. There were no significant differences in preoperative clinical characteristics between the flange group and the suture group. However, the proportion of IOL exchange was higher in the flange group compared to the suture group (48.4% vs. 20.6%, *P* = 0.018). Additionally, the mean operation time in the flange group was shorter than that of the suture group (90.8 ± 19.9 vs. 110.0 ± 32.9 min, *P* = 0.007).

**Table 1:** Clinical characteristics of patients. IOL, intraocular lens.

	Total (n = 65)	Flange group (n = 31)	Suture group (n = 34)	*P*
Male sex, n (%)	49 (75.4)	25 (80.6)	24 (70.6)	0.347*
Age (years), mean ± SD	68.5 ± 10.8	68.6 ± 8.5	68.3 ± 12.6	0.923†
Follow-up period (months), mean ± SD	14.0 ± 6.6	12.8 ± 5.7	15.1 ± 7.3	0.129‡
Indications for surgery, n (%)				
Aphakia	19 (29.2)	9 (29.0)	10 (29.4)	0.874**
Dislocated intraocular lens	37 (56.9)	17 (54.8)	20 (58.8)	
Subluxated crystalline lens	9 (13.8)	5 (16.1)	4 (11.8)	
Right eye, n (%)	27 (41.5)	13 (41.9)	14 (41.2)	0.951*
Intraocular pressure (mmHg), mean ± SD	17.2 ± 6.3	16.2 ± 4.2	18.1 ± 7.7	0.553‡
Axial length (mm), mean ± SD	24.0 ± 1.4	23.8 ± 1.1	24.2 ± 1.6	0.213†
IOL power (diopter), mean ± SD	19.9 ± 3.5	20.5 ± 3.0	19.1 ± 3.9††	0.128†
IOL exchange, n (%)	22 (33.8)	15 (48.4)	7 (20.6)	0.018*
Operation time (minutes), mean ± SD	100.9 ± 28.9	90.8 ± 19.9	110.0 ± 32.9	0.007†

*Chi-square test. †Independent t-test. ‡Mann–Whitney U test. **Fisher’s exact test. ††Analysis was conducted excluding the seven eyes that underwent scleral fixation using IOLs implanted in previous surgery without available IOL power information.

### Visual and refractive outcomes

The flange group showed significantly better mean uncorrected visual acuity (UCVA) than the suture group at 2 months and 6 months after surgery (Table 2). Until 1 month postoperatively, there were no significant differences between the two groups. However, at 6 months postoperatively, the flange group exhibited better UCVA compared to the suture group (0.251 [20/36 in Snellen] ± 0.328 vs. 0.418 [20/52 in Snellen] ± 0.339 logMAR, *P* = 0.041). Within each group, the mean UCVA significantly improved from one month postoperatively (both *P* < 0.001).

**Table 2:** Visual and Refractive Outcomes.

	Total (n = 65)	Flange group (n = 31)	Suture group (n = 34)	*P*
Preoperative				
UCVA (logMAR)	1.292 ± 0.697	1.248 ± 0.672	1.331 ± 0.727	0.637*
BCVA (logMAR)	0.320 ± 0.402	0.266 ± 0.239	0.369 ± 0.506	0.307*
Spherical equivalent (D)	6.72 ± 5.53	7.24 ± 5.37	6.24 ± 5.72	0.472*
Refractive astigmatism (D)	− 0.61 ± 0.96	− 0.46 ± 0.80	− 0.74 ± 1.07	0.236*
Postoperative, 1 month				
UCVA (logMAR)	0.484 ± 0.355	0.425 ± 0.346	0.538 ± 0.360	0.203*
BCVA (logMAR)	0.297 ± 0.296	0.276 ± 0.299	0.316 ± 0.297	0.596*
Spherical equivalent (D)	− 1.03 ± 1.09	− 0.71 ± 0.94	− 1.33 ± 1.15	0.019*
Refractive astigmatism (D)	− 1.32 ± 1.00	− 1.14 ± 0.79	− 1.48 ± 1.16	0.175*
Postoperative, 2 month				
UCVA (logMAR)	0.342 ± 0.285	0.266 ± 0.244	0.411 ± 0.305	0.048†
BCVA (logMAR)	0.196 ± 0.225	0.158 ± 0.193	0.231 ± 0.249	0.194*
Spherical equivalent (D)	− 1.06 ± 1.08	− 0.73 ± 0.92	− 1.36 ± 1.15	0.018*
Refractive astigmatism (D)	− 1.30 ± 0.94	− 1.20 ± 0.79	− 1.38 ± 1.05	0.441*
Postoperative, 6 month				
UCVA (logMAR)	0.327 ± 0.334	0.251 ± 0.328	0.418 ± 0.339	0.041*
BCVA (logMAR)	0.149 ± 0.203	0.125 ± 0.213	0.171 ± 0.195	0.367*
Spherical equivalent (D)	− 1.11 ± 1.07	− 0.74 ± 0.93	− 1.44 ± 1.09	0.007*
Refractive astigmatism (D)	− 1.30 ± 0.93	− 1.18 ± 0.73	− 1.41 ± 1.09	0.316*

UCVA, uncorrected visual acuity; BCVA, best corrected visual acuity; logMAR, logarithm of the minimum angle of resolution; D, diopter. *Independent t-test. †Mann–Whitney U test.

At 6 months after surgery, both the flange group and the suture group demonstrated significant improvements in best-corrected visual acuity (BCVA) compared to preoperative values (*P* = 0.023 and *P* = 0.018, respectively). There were no significant between-group differences in mean BCVA at any time point.

Of note, the flange group exhibited a spherical equivalent closer to targeted emmetropia compared to the suture group at 1 month, 2 months, and 6 months after surgery (*P* = 0.019, *P* = 0.018, and *P* = 0.007, respectively). At 6 months after surgery, the mean spherical equivalent in the flange group was − 0.74 ± 0.93 diopters, while that in the suture group was − 1.44 ± 1.09 diopters.

Regarding ocular residual astigmatism, J45 showed statistically significant differences from 1 to 6 months postoperatively in the suture group (*P* = 0.012) (Supplementary Table S1). However, in the flange group, J45 did not show a significant difference between 1 and 6 months after surgery (*P* = 0.335). There were no significant between-group differences in both J0 and J45 at any time point after surgery. Similarly, no significant difference in corneal astigmatism was observed between groups at any time point, and there was no significant difference up to 6 months postoperatively compared to preoperative values (Supplementary Table S2).

### Postoperative complications

No intraoperative complications developed in either group. The postoperative complications are presented in Table 3. We observed no instances of iris capture in the flange group, while iris capture occurred in ten eyes, accounting for 29.4% of the suture group (*P* < 0.001). Decentered IOL did not occur in the flange group, but it occurred in eight eyes (23.5%) in the suture group (*P* = 0.005). Of total, the IOL was dropped into the vitreous cavity in only one eye in the suture group. Although there was no statistical significance between the two groups, all three eyes (4.6%) that required reoperation due to IOL instability belonged to the suture group.

**Table 3:** Postoperative Complications.

	Flange group (n = 31)	Suture group (n = 34)	*P*
Iris capture of IOL, n (%)	0 (0)	10 (29.4)	< 0.001*
Decentered IOL, n (%)	0 (0)	8 (23.5)	0.005*
Reoperation due to IOL instability, n (%)	0 (0)	3 (8.8)	0.240*
Flange or suture exposure, n (%)	4 (12.9)	7 (20.6)	0.409†
Transient IOP elevation, n (%)	9 (29.0)	13 (38.2)	0.434†
Secondary glaucoma, n (%)	0 (0)	1 (2.9)	> 0.999*
Hypotony (< 10 mmHg), n (%)	3 (9.7)	3 (8.8)	> 0.999*
Cystoid macular edema, n (%)	3 (9.7)	4 (11.8)	> 0.999*
Bullous keratopathy, n (%)	2 (6.5)	2 (5.9)	> 0.999*

IOL, intraocular lens; IOP, intraocular pressure. *Fisher’s exact test. †Chi-square test.

In terms of knot exposure, suture exposure occurred in seven eyes (20.6%) in the suture group, while flange exposure occurred in four eyes (12.9%) in the flange group (*P* = 0.409). The most common postoperative complication among all cases was transient elevation of IOP (22 eyes, 33.8%) with discontinuation of IOP-lowering medication within 1 month postoperatively. On the other hand, hypotony occurred in six eyes (9.2%) in all cases and resolved spontaneously within two weeks after surgery. During the follow-up period, cystoid macular edema and bullous keratopathy did not show a significant difference between the two groups. In the flange group, no opacification was observed in the fourteen Akreos IOLs.

## Discussion

In the present study, we observed that the double-flanged (four-flanged) sutureless scleral fixation technique resulted in a smaller myopic shift, leading to improved UCVA, compared to conventional sutured scleral fixation. Furthermore, the double-flanged technique demonstrated reduced operation time and decreased postoperative complications such as iris capture and IOL decentration, which is consistent with previous studies reporting no instances of iris capture after four-point sutured scleral fixation^13,14^. Also, there was a significant change in J45 between 1 and 6 months postoperatively in the suture group, indicating a noticeable alteration in IOL astigmatism from 1 to 6 months after conventional fixation. Although there is limited literature comparing the Canabrava technique with other techniques, previous studies have reported comparable or even better visual outcomes and IOL stability with four-point fixation compared to two-point fixation^14,15^.

Notably, the double-flanged technique provided enhanced stability of the IOL compared to the conventional technique. This might have contributed to the favorable outcomes, including reduced IOL capture, decentration, and myopic shift. Since both methods positioned the aspherical IOLs at 2.0 mm from the limbus, there may be other factors contributing to the difference in IOL stability. First, the main distinction between the two techniques lies in the number of fixation points. The double-flanged technique allows for the fixation of the IOL at four points, effectively doubling the number of fixation points compared to the conventional technique. In the event of thread loosening after surgery, the remaining three flanges of the double-flanged technique could ensure a more stable fixed position of the IOL. In contrast, in two-point fixation, the IOL position might not be sustained if only one thread remains intact. If the thread breaks, the double-flanged technique would offer the advantage of easily removing the broken thread, allowing for the re-threading of the IOL into position within the eye without the need for a suture procedure. Additionally, our technique positions four flanges at wider 90-degree intervals in four directions, unlike the original method that secures the artificial lens at both ends in two directions. We hypothesized that this modification would result in decreased IOL tilt by providing a more secure fixation of the IOL.

Second potential factor could be the difference in IOL design between the two methods. The conventional technique used a three-piece IOL with two C-shaped polymethylmethacrylate (PMMA) haptics, while the double-flanged technique utilized a single-piece acrylic IOL with four closed-loop haptics. Although both surgical methods securely fix the haptics to the sclera, the thin diameter (approximately 0.15 mm) of the PMMA haptics may allow for relatively free movement of the optic due to their weak resistance to external forces^16^, compared to the thicker acrylic haptics. In addition to the inherent strength of the haptic itself, the haptic-optic junction of the three-piece IOL is more pliable than that of single-piece acrylic IOL^17^.

Another factor that could impact IOL stability is the difference in thickness and strength between the 10-0 and 5-0 polypropylene threads. The use of 10-0 suture carries a risk of loosening or breakage^18,19^, which may result in an inability to achieve sufficient tightness with a knot^20^. A biomechanical study demonstrated that the 1.0 mm 5-0 polypropylene flange had greater strength than the 10-0 polypropylene suture^20^. Thus, utilizing four 5-0 polypropylene flanges in the double-flanged technique would provide a more robust and stable fixation of the IOL to the sclera, surpassing the fixation achieved with two 10-0 polypropylene sutures.

Consistent with a previous study^19^, both groups exhibited a postoperative myopic shift, but the flange group showed significantly less myopic shift compared to the suture group. The enhanced stability resulting from the firm fixation of the posterior chamber IOL likely contributed to the prevention of forward movement, thereby reducing myopic shift, improving UCVA, and avoiding iris capture^6,9^. In cases of routine in-the-bag IOL implantation, the optic of a three-piece IOL is hindered from moving forward towards the iris plane by an appropriately sized anterior continuous curvilinear capsulorhexis. However, in eyes without capsular support, maintaining the position of the IOL further behind the limbus can help prevent myopic shift and iris capture^15,21^. Therefore, in future procedures, we plan to adjust the surgical method to fixate it at the 2.5 mm from the limbus.

The double-flanged technique simplifies the surgery and reduces procedure time by eliminating the need for knots and flaps, and only involves creating flanges using cautery^12^. In the flange group, the mean operation time recorded in the surgical records was approximately 90 min, which may seem long. However, this duration included not only the time required for IOL fixation but also the time for removing the crystalline lens material, the existing IOL, and performing vitrectomy. While we did not measure the time taken to fix the intraocular lens, preventing an accurate comparison of the time required for intraocular lens fixation, the double-flanged method showed a reduction of approximately 20 min in IOL fixation time compared to the conventional method. Moreover, one of the most advantageous aspects was the easier manipulation of 5–0 polypropylene compared to 10-0 polypropylene.

In the case of surgery performed by beginners, the Yamane technique may result in haptic kinking or breakage, necessitating the removal of the IOL and insertion of a new one during the surgery^11^. However, with the double-flanged technique, there is minimal risk of thread breakage or kinking since the thread is flexible. Even if the thread becomes dislodged during surgery, it can be easily removed and repositioned within the eye. Therefore, depending on the surgeon, handling unexpected situations during surgery might be easier with the double-flanged technique, leading it to be perceived as a safer alternative compared to the Yamane technique.

The main drawback we encountered with the double-flanged technique was its incompatibility with a two-haptic IOL, which required IOL exchange unless a 4-eyelet IOL had been previously used. Thus, the most troublesome aspect was that a larger incision was required for removing the one-piece PMMA IOL. Since incorrect marking can lead to IOL decentration, it is crucial to exercise caution during the marking process. We also experienced some cases of flange exposure, which were managed with simple conjunctival sutures or electrocoagulation. In contrast to the recently published 5-year results by Canabrava^22^, we did not observe any instances of internalized flanges. This difference might be attributed to the difference in the size of the flange used in our study^20^. Since the Artis PL E hydrophobic acrylic IOL became unavailable, the Akreos AO60 hydrophilic acrylic IOL had to be used as an alternative option. However, no opacification of Akreos IOLs was observed, which is consistent with a previous report^15^. If possible, using a hydrophobic IOL would be preferable, as there have been reports of opacification occurring with Akreos IOL^23^.

There are several limitations to this study. First, due to the lack of measurement of IOL tilt, we were unable to analyze the extent and timing of IOL tilt occurrence. Secondly, our study had a short-term follow-up and a small sample size due to the utilization of the recently published Canabrava technique. Thirdly, the surgical method was not randomized but based on surgeon’s preference, introducing potential bias. Therefore, a randomized clinical trial with larger sample sizes and a long-term follow-up period might be necessary to compare various methods with the double-flanged technique. Nonetheless, this study holds significance as it is the first to compare the double-flanged technique with another technique, including a statistically comparable number of patients, and the follow-up period was sufficient to investigate short-term outcomes and postoperative complications. We have plans to present various modifications related to the Canabrava technique and comparative study with the widely used Yamane technique in the future.

In conclusion, the double-flanged technique could offer several advantages, including the prevention of iris capture and IOL decentration, as well as a reduction in myopic shift through enhanced IOL stability. This technique facilitated a faster and easier achievement of a stable IOL position and resulted in favorable visual outcomes. By combining the stability of the four-point scleral fixation with the technical simplicity of the flange technique, this approach holds the potential to improve surgical outcomes.

## Methods

This study was approved by the Institutional Review Board (IRB No. Hallym 2023-05-019) of Hallym University Sacred Heart Hospital and was conducted in accordance with the tenets of the Declaration of Helsinki. Patient consent was waived due to the retrospective nature of the study and the minimal level of risk arising from the study.

### Subjects

The medical records of patients who underwent modified double-flanged sutureless scleral fixation or conventional sutured scleral fixation at Hallym University Sacred Heart Hospital between March 2021 and December 2022 were retrospectively reviewed. Indications for surgery included aphakia due to complicated cataract surgery, dislocated IOL, and subluxated crystalline lens. Eyes with open globe injury, previous retinal disease, or previous extracapsular cataract extraction were excluded. We also excluded eyes undergoing IOL exchange combined with the removal of PMMA IOL. The patients with a postoperative follow-up period of less than 6 months were excluded. If both eyes of the same patient met all the study criteria, one eye was randomly selected. Based on the surgical technique, all eligible eyes were assigned to either the flange group or the suture group. We routinely aimed for emmetropia using the SRK/T formula as the standard.

### Surgical technique

All surgeries were performed by experienced one vitreoretinal surgeon (S.K.). The selection of the surgical technique was determined by the surgeon’s preference. A standard 3-port, 25-gauge pars plana vitrectomy (Constellation Vitrectomy System; Alcon Laboratories, Texas, USA) was performed under retrobulbar anesthesia. In cases of IOL dislocation, if necessary, the existing IOL was exchanged. Phacoemulsification was performed for subluxated crystalline lenses. Regardless of eye laterality and surgical technique, a 2.8 mm clear corneal incision was made at 11 o’clock.

### Modified double-flanged technique

The original procedure had been described by Canabrava^7,12^. Two types of foldable IOLs with four eyelets were utilized: the Artis PL E hydrophobic acrylic IOL (Cristalens Industrie, Lannion, France) or the Akreos AO60 hydrophilic acrylic IOL (Bausch and Lomb, New Jersey, USA). To simplify the procedure of the original technique, a foldable IOL was inserted using an injector through a clear corneal incision of 2.8 mm, and the needle, already threaded with 5–0 polypropylene (Ethicon, New Jersey, USA), was passed through the eyelets of trailing haptics within the eye (see Video, Supplemental Digital Content).

**Figure 1:**
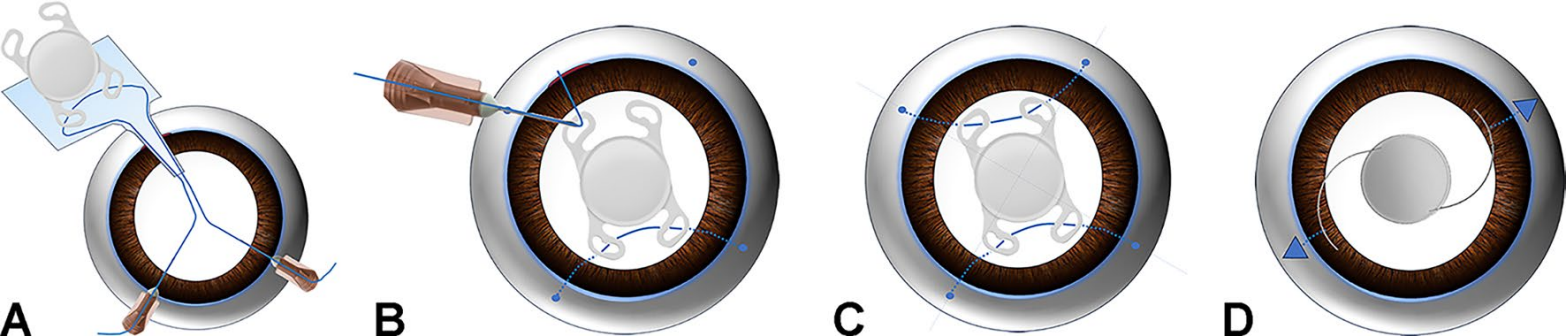
Modified double-flanged technique (**A**–**C**) and conventional sutured scleral fixation (**D**). (**A**) The foldable intraocular lens (IOL) threaded through the leading haptics was inserted using an injector through a 2.8 mm corneal incision. (**B**) For the externalization of the second thread, a 26-gauge needle, already threaded with 5–0 polypropylene, passed from bottom to top through the trailing haptic. (**C**) The IOL was fixated using four flanges positioned at 1, 4, 7, and 10 o'clock. (**D**) The IOL was fixated using the ab externo technique at the 2, and 8 o’clock positions.

Using the Mendez degree gauge with a bores axis marker (Katena, New Jersey, USA), markings were made on the limbus along two straight lines at a right angle, corresponding to the 1 and 7 o'clock positions, as well as the 4 and 10 o'clock positions. Before loading the IOL, the first 5-0 polypropylene was passed through eyelets of two leading haptics, which would be located at the 4 and 7 o’clock. The docking method, identical to the original technique, was performed^12^. The IOL was inserted using an injector through the corneal incision (Fig. 1A). A 26-gauge needle, already threaded by 5-0 polypropylene, was inserted through the 10 o’clock sclerotomy and passed into the haptic from the bottom to the top (Fig. 1B). For the final docking at the 1 o’clock, the 5-0 polypropylene tip was externalized through the corneal incision and docked into the 26-gauge needle outside the eye. After adjusting the IOL position, all flanges were completed and positioned on the sclera (Fig. 1C).

### Conventional ab externo technique

Conjunctival peritomy and limbal-based triangular scleral flap were made after marking two points at the 2 o'clock and 8 o'clock positions (Fig. 1D). A double-arm 10-0 polypropylene suture was passed through both scleral patches 2.0 mm from the limbus using a 26-gauge needle. The thread was taken out through the corneal incision and tied to the PMMA haptics of Sensar AR40e (Abbott Medical Optics, California, USA) three-piece IOL. The IOL was positioned at the center of the eye. Scleral sutures were performed to fix the IOL and covered with a scleral flap. The scleral flaps and the conjunctiva were closed.

### Data collection

Ophthalmic examination included the measurement of UCVA and BCVA, intraocular pressure, axial length, slit-lamp biomicroscopy, and dilated fundus examination. The preoperative and postoperative refractive errors were measured by manifest refraction. The operation time was defined as the duration from the first incision to the completion of the procedure, as recorded in the surgical records.

Since direct measurement of IOL tilt using equipment such as Scheimpflug image or anterior optical coherence tomography was not performed, the degree of IOL tilt was indirectly evaluated through ocular residual astigmatism. To calculate ocular residual astigmatism, the power vectors expressed in terms of magnitude (the spherical equivalent), J0 (Cartesian astigmatism), and J45 (oblique astigmatism) were utilized^24^. First, refractive astigmatism in polar form at the spectacle plane was converted to the corneal plane^25^, and then transformed into vector form^24,26^. Similarly, corneal astigmatism in polar form was also transformed into vector form. As a result, the ocular residual astigmatism was calculated by subtracting the vector form of corneal astigmatism from the vector form of refractive astigmatism, which refers to the components of IOL. To enhance intuitive comprehension of the magnitude of IOL astigmatism, the power vector notation was converted to the cylinder form.

### Statistical analysis

Continuous variables were presented as means ± standard deviation, and categorical variables were expressed as numbers and proportions. Visual acuity was converted to the logMAR for statistical analysis. The logMAR values corresponding to counting fingers and hand motion were replaced with 2.10 and 2.40, respectively^27^. The chi-square test was used to compare categorical variables between the two groups. If the expected values were less than 5, Fisher’s exact test was employed. To compare continuous variables, an independent t-test, or a nonparametric test (Mann–Whitney U) was utilized. The paired t-test or Wilcoxon signed-rank test was used to compare paired measurements associated with visual and refractive outcomes within each group. A *P* value of less than 0.05 was considered statistically significant. All analyses were conducted using SPSS (version 26, IBM Corporation, Armonk, New York, USA).

### Supplementary Information

Supplementary Video 1.

Supplementary Tables.

## Data Availability

The clinical datasets used in current study are not publicly available due to privacy constraints. The data can be requested for sharing for peer-review or research purposes by contacting Soonil Kwon (magicham@daum.net).
