# Supplementary material for: Comparison of outcomes between modified double-flanged sutureless scleral fixation and conventional sutured scleral fixation
Source: Sci Rep. 2024 Jul 12;14:16111. doi: 10.1038/s41598-024-66762-y (PMC11245608; doi:10.1038/s41598-024-66762-y)
Supplement: Supplementary file 2 — Supplementary Tables. [file 41598_2024_66762_MOESM2_ESM.pdf]

Comparison of Outcomes between Modified Double-flanged Sutureless Scleral Fixation and Conventional Sutured Scleral Fixation

Jinsoo Kim[1], Phil Young Lee[2], Min Seon Park[1], Bum-Joo Cho[1], Soonil Kwon[1*]

[1]Department of Ophthalmology, Hallym University Sacred Heart Hospital, Hallym University College of Medicine, Anyang, Korea

[2]Department of Ophthalmology, Veterans Health Service Medical Center, Seoul, Korea

Supplementary table S1. Ocular Residual Astigmatism. J_0, the vertical Jackson's cross cylinder; J_{45}, the oblique Jackson's cross cylinder; N/A, not applicable. * *P* values were calculated by comparing the values at those time points to the values at the 1-month postoperative time point. †*P* values were calculated using independent t-test or Mann-Whitney U test.

	Total (n = 65)		Flange group (n = 31)		Suture group (n = 34)		*P* value between groups†
	Mean ± SD	*P* value*	Mean ± SD	*P* value*	Mean ± SD	*P* value*	
Postoperative, 1 month							
J_0, power vector	-0.09 ± 0.42	N/A	-0.06 ± 0.31	N/A	-0.12 ± 0.51	N/A	0.990
J_{45}, power vector	0.21 ± 0.56	N/A	0.13 ± 0.53	N/A	0.27 ± 0.59	N/A	0.318
Plus cylinder, diopter	1.13 ± 0.95	N/A	0.95 ± 0.81	N/A	1.30 ± 1.04	N/A	N/A
Postoperative, 2 month							
J_0, power vector	-0.06 ± 0.39	0.434	-0.07 ± 0.28	0.792	-0.05 ± 0.48	0.310	0.511
J_{45}, power vector	0.14 ± 0.48	0.188	0.05 ± 0.49	0.246	0.22 ± 0.46	0.458	0.090
Plus cylinder, diopter	0.99 ± 0.80	N/A	0.88 ± 0.72	N/A	1.09 ± 0.87	N/A	N/A
Postoperative, 6 month							
J_0, power vector	-0.11 ± 0.41	0.841	-0.07 ± 0.24	0.799	-0.14 ± 0.52	0.905	0.958
J_{45}, power vector	0.03 ± 0.47	0.009	0.05 ± 0.54	0.335	0.01 ± 0.39	0.012	0.715
Plus cylinder, diopter	1.01 ± 0.75	N/A	0.93 ± 0.75	N/A	1.08 ± 0.75	N/A	N/A

Supplementary table S2. Corneal Astigmatism. J_0, the vertical Jackson's cross cylinder; J_{45}, the oblique Jackson's cross cylinder; N/A, not applicable. * *P* values were calculated by comparing the values at those time points to the values at the preoperative time point. †*P* values were calculated using independent t-test or Mann-Whitney U test.

	Total (n = 65)		Flange group (n = 31)		Suture group (n = 34)		*P* value between groups†
	Mean ± SD	*P* value*	Mean ± SD	*P* value*	Mean ± SD	*P* value*	
Preoperative							
J_0, power vector	0.06 ± 0.67	N/A	-0.09 ± 0.61	N/A	0.20 ± 0.70	N/A	0.083
J_{45}, power vector	-0.05 ± 0.50	N/A	-0.05 ± 0.34	N/A	-0.05 ± 0.62	N/A	0.962
Plus cylinder, diopter	1.37 ± 0.98	N/A	1.15 ± 0.80	N/A	1.57 ± 1.09	N/A	N/A
Postoperative, 1 month							
J_0, power vector	0.10 ± 0.65	0.551	0.04 ± 0.59	0.049	0.15 ± 0.71	0.638	0.478
J_{45}, power vector	-0.19 ± 0.54	0.054	-0.23 ± 0.53	0.075	-0.16 ± 0.56	0.316	0.590
Plus cylinder, diopter	1.42 ± 0.99	N/A	1.40 ± 0.84	N/A	1.45 ± 1.13	N/A	N/A
Postoperative, 2 month							
J_0, power vector	-0.001 ± 0.60	0.252	-0.06 ± 0.60	0.658	0.05 ± 0.60	0.071	0.477
J_{45}, power vector	-0.14 ± 0.46	0.179	-0.15 ± 0.50	0.316	-0.13 ± 0.42	0.380	0.861
Plus cylinder, diopter	1.28 ± 0.83	N/A	1.32 ± 0.85	N/A	1.24 ± 0.83	N/A	N/A
Postoperative, 6 month							
J_0, power vector	-0.02 ± 0.60	0.167	-0.07 ± 0.59	0.755	0.03 ± 0.62	0.057	0.540
J_{45}, power vector	-0.08 ± 0.44	0.622	-0.15 ± 0.50	0.326	-0.02 ± 0.38	0.794	0.245
Plus cylinder, diopter	1.26 ± 0.82	N/A	1.33 ± 0.82	N/A	1.19 ± 0.82	N/A	N/A
